# Relationship between job burnout and somatic diseases: a network analysis

**DOI:** 10.1038/s41598-020-75611-7

**Published:** 2020-10-28

**Authors:** Roland von Känel, Mary Princip, Sarah A. Holzgang, Walther J. Fuchs, Marc van Nuffel, Aju P. Pazhenkottil, Tobias R. Spiller

**Affiliations:** 1 Department of Consultation-Liaison Psychiatry and Psychosomatic Medicine, University Hospital Zurich, University of Zurich, Culmannstrasse 8, 8091 Zurich, Switzerland; 2 Burnout Protector GmbH, Küsnacht, Switzerland; 3 Digiboo GmbH, Zurich, Switzerland; 4 DU DA-Data & Commtech by Farner, Zurich, Switzerland; 5 Cardiac Imaging, Department of Nuclear Medicine, University Hospital Zurich, Zurich, Switzerland; 6 Department of Cardiology, University Hospital Zurich, Zurich, Switzerland

**Keywords:** Psychology, Cardiology, Health care, Medical research, Risk factors

## Abstract

Job burnout, characterized by feelings of exhaustion, cynicism and reduced personal efficacy, has been defined as a risk state for the development of diseases, but its association with somatic diseases is underexplored. Study participants were 5671 respondents (mean age 44.1 years, range 18–70; 38.6% men) to an online survey of job burnout, using a mobile health web application. Respondents provided data on sociodemographic characteristics, symptoms of burnout, measured with the Maslach Burnout Inventory-General Survey, depressive symptoms, measured with the Profile of Mood States, and 11 categories of somatic diseases. Adjusting for age, sex, educational level, depressive symptoms, and all disease categories included, network analysis showed a significant association of high exhaustion with “high blood pressure” and a category of “other chronic somatic diseases”. Low personal efficacy showed a significant association with “chronic lung diseases”. In network analysis, clinically relevant depressive symptoms were also significantly associated with “high blood pressure”, “other chronic somatic diseases” and “skin diseases”. Logistic regression analysis confirmed these associations. Burnout dimensions were significantly associated with an increased risk for somatic diseases, independently of sociodemographic factors and clinically relevant depressive symptoms. This relationship seems particularly evident for the exhaustion dimension of job burnout.

## Introduction

Job burnout is conceptualized as a psychological syndrome that results from chronic stress at work^1^. The key dimensions exhaustion (EX), cynicism (CYN), and reduced personal efficacy (PE) can be assessed with the Maslach Burnout Inventory (MBI), the most frequently used self-rating instrument and standard tool for burnout research^2^. For research purposes, there is no definite consensus as to whether burnout should be treated as a multidimensional or unidimensional construct, and whether categorical or continuous data should be used^3^. Yet, it can be argued that unidimensional views of burnout may overestimate the exhaustion experience and that physicians prefer cut-off values for burnout scores to distinguish patients with clinically relevant burnout^2,4,5^.

Burnout is not classified as a disease, but as a risk factor of such, which may affect health status and increase health service use^1,6^. However, the link between burnout and the risk of somatic diseases remains underexplored^7^. Arguably, the most consistent finding is an association of burnout with cardiovascular disease (CVD) and cardiovascular risk factors, including high blood pressure (BP)^8–10^. These associations are stronger with more severe burnout, predominantly observed for the EX dimension of burnout, and also with adjustment for depressive symptoms^11^. The latter is of conceptual importance, as there is an ongoing debate among researchers about the extent to which burnout and depression are overlapping constructs^11,12^. Therefore, when investigating the hypothesis of an independent association between burnout and its dimensions with physical health outcomes, it is important to make adjustment for depressive symptomatology.

We investigated the association of high EX, high CYN and low PE with somatic diseases in users of a mobile health web application^13^ performing network analysis, a novel approach in this field of research. The used network analytical method allows to investigate the association of two variables, while simultaneously controlling for the effect of all other variables in datasets with multiple variables. Sociodemographic factors and depressive symptoms were taken into account as covariates in statistical models.

## Methods

### Study participants

Study participants were consecutive respondents to an online survey of burnout risk performed between 11/2016 and 9/2019. A campaign was launched via Medinside, Winsider AG, Winterthur, Switzerland, an open access portal for the health care sector, particularly recruiting physicians, as they show high prevalence of burnout^14^. Data were collected with Burnout Protector, a previously described mobile health web application^13^. Age below 18 was the only exclusion criterion. We did not consider data of respondents above 70 years, the age of the latest official retirement in Switzerland. We identified participants which answered the survey twice or more by their assigned user identification. We included only their initial answer in the analysis.

This study was carried out in accordance with the Swiss Human Research Ordinance (i.e., under strict confidentiality and privacy, with coding of health-related personal data). Ethical authorization was not required, as this study does not fall within the scope of the Swiss Human Research Act. Nevertheless, all data was collected in accordance with scientific standards specified by Switzerland’s Federal Act on Research Involving Human Beings^15^ and in compliance with the national ethical guidelines on the handling of personal data in the field of medicine issued by the Federal Data Protection Commissioner of Switzerland^16^. All respondents provided informed consent to their anonymized data being used for research^13^.

### Measures

Respondents indicated their age, sex, and highest level of education. Somatic diseases were inquired with the closed-ended question “Are you suffering from any of the following somatic diseases?” referring to 11 categories. These were high blood pressure, high cholesterol, diabetes, stroke, myocardial infarction, chronic heart failure, chronic lung disease, chronic liver disease, chronic renal disease, chronic skin disease, and a residual category of “other chronic somatic disease”. Participants could select the response option “other chronic somatic disease” if they suffered from a chronic disease which was not among the ten specified categories on the shortlist.

The 16-item MBI-General Survey (MBI-GS) was applied to assess EX (5 items), CYN (5 items) and PE (6 items)^17,18^. Each item is rated on a 7-point Likert scale from 0 (“never”) to 6 (“daily”). In accordance with a previous population-based study from Finland, showing associations of both the total score of the MBI-GS and each of its dimensions with physical health outcomes, we used a mean score of 3.50 or higher across all items of a subscale to define high EX, high CYN and high lack of PE^8^. This categorization means that the three burnout dimensions are in the severe range (3.50–6) when symptoms of either EX, CYN or lack of PE are experienced about once a week or daily^8^. In a previous study on outpatients with work-related psychological problems, the MBI-GS showed the highest sum of sensitivity (78%) and specificity (48%) with a cutoff point of 3.50 on the EX subscale, which was recommended to minimize false negative burnout cases^19^. Moreover, individuals working under highly demanding conditions who scored above 3.50 points on the EX subscale of the MBI-GS showed greater physiological dysregulation, evidenced by lower heart rate variability, higher cortisol levels and higher blood pressure, than their counterparts with lower EX scores^10^. A 14-item subscale of the Profile of Mood States (POMS) was used to assess the severity of depressive symptoms^20,21^. Each item is rated on a 5-point Likert scale from 0 (“not at all”) to 4 (“extremely”). Clinically relevant depressive symptoms were defined by a score of 14 or higher^22^.

### Statistical analysis

For network analysis and following logistic regression, categorical variables for high vs. low scores of each burnout dimension, clinically relevant vs. non-relevant depressive symptoms and high versus low educational level were used^13^. Age was dichotomized at a threshold of 45 years, as EX was shown to be predictive of increased all-cause mortality in employees under 45 years of age^23^. In all analyses, the included variables were dichotomous. The network analysis was performed using the eLASSO procedure^24^. Conceptually, for each node in the network, a regularized logistic regression is performed, with the given node as dependent variable (outcome) and all other included variables as independent variables (predictors)^24^. The chosen eLASSO procedure is a L1-regularization regression procedure based on LASSO (least absolute shrinkage and selection operator)^25^ and adapted to analyze networks of dichotomous variables. In such a procedure, the penalization causes small estimates to shrink to zero. Hence, adjustment of individual p values is not necessary (for more details see^24^). In a resulting network, nodes and edges represent the variables and the associations between variables, respectively, whereas the associations correspond to parameter estimates of a logistic regression model, whose magnitude can be transformed to odds ratio. In a second step, we investigated the associations of interest revealed by network analysis using multivariable, non-regularized logistic regression. In these models, the dependent variable (outcome) was a somatic disease of interest and all other variables also included in the network analysis were again used as independent variables. We tested no interaction terms. The variance inflation factor for all included variables in all tested models was below 3, thus indicating no multicollinearity. For the logistic regressions, all variables were entered collectively. The event rate of the included variables, their coding, the detailed results of the analyses (incl. goodness-of-fit indices and results of the significance testing of the individual parameters) are presented in the supplementary materials (Supplementary Tables S1–S4). All analyses were carried out in R (version 3.6.1)^26^. Estimation and visualization of the network was carried out using R-Package Bootnet (version 1.2.4)^27^. The analytic file (“Syntax”) is provided as supplementary information.

## Results

**Figure 1:**
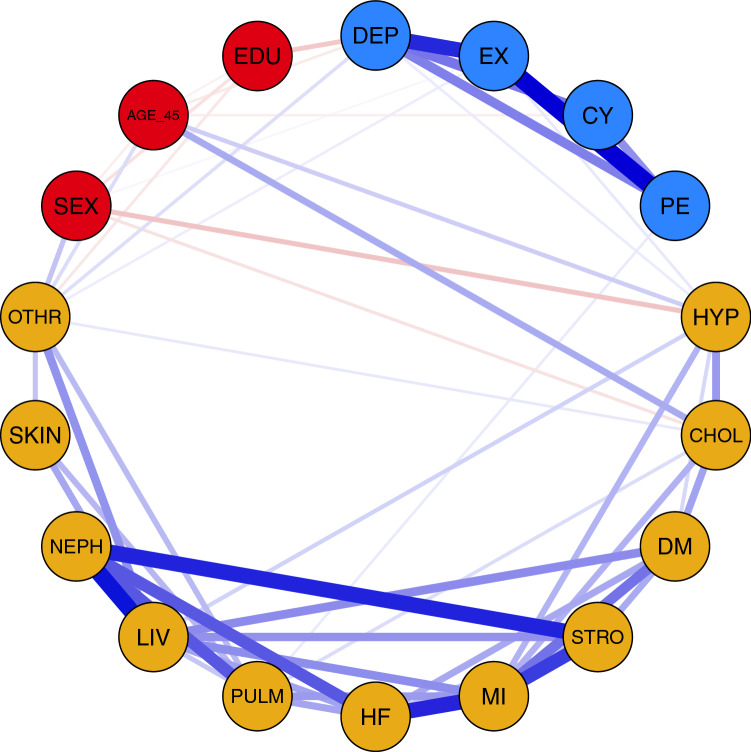
Network of the burnout dimensions (EX, CYN, PE), symptoms of depression (DEP), sex, age, level of education and categories of diseases. Nodes represent burnout dimensions (high EX, high CYN, low PE), clinically relevant symptoms of depression (DEP), sex (male sex as the reference category), age, high level of education and categories of diseases. Blue edges indicate positive odds ratios, red edges indicate negative odds ratios. The thickness of the edge represents the magnitude of the odds ratio. Red nodes represent demographic variables, blue nodes psychological variables and yellow nodes somatic diseases. *SEX* sex, *Age_4*5 dichotomized age (< 45 years vs. ≥ 45 years), *EDU* education, *DEP* depressive symptoms, *EX* exhaustion, *CYN* cynicism, *PE* personal efficacy, *HYP* high blood pressure, *CHOL* high cholesterol, *DM* diabetes, *STRO* stroke, *MI* myocardial infarction, *HF* chronic heart failure, *PULM* chronic lung disease, *LIV* chronic liver disease, *NEPH* chronic renal disease, *SKIN* chronic skin disease, *OTHR* other chronic somatic disease.

Table 1 shows the characteristics of the 5671 study participants. Figure 1 shows the results of the network analysis. The estimated network was sparse. Most edges were between somatic diseases, and between psychological variables, whereas only few edges were found between somatic diseases and psychological variables. Among them, high EX was associated with high BP and other chronic diseases, and low PE was associated with chronic lung diseases. Figure 1 additionally shows that clinical depressive symptoms were associated with high BP, skin diseases and other chronic diseases. With regard to demographic variables, education was associated with symptoms of clinical depression, sex was positively related with high EX, but negatively with high BP and high cholesterol.

**Table 1:** Characteristics of the 5671 study participants.

Male sex, n	2190 (38.7)
Age, years	44.1 (11.7)
High educational level^a^, n	3287 (58.0)
High blood pressure, n	1066 (18.9)
High cholesterol, n	907 (16.0)
Diabetes, n	209 (3.7)
Stroke, n	135 (2.4)
Myocardial infarction, n	148 (2.6)
Chronic heart failure, n	147 (2.6)
Chronic lung disease, n	339 (6.0)
Chronic liver disease, n	155 (2.7)
Chronic renal disease, n	132 (2.3)
Chronic skin disease, n	665 (11.8)
Other chronic somatic disease, n	1028 (18.1)
High exhaustion, n	3154 (55.6)
High cynicism, n	587 (10.4)
Low personal efficacy, n	1932 (34.1)
Clinically relevant depressive symptoms, n	3473 (61.2)

Values are given as mean with SD for age and absolute numbers with the percentage share of the total sample.

^a^ Defined as university degree, school leaving examination, or vocational school certificate.

All associations between psychological variables and somatic outcomes were confirmed by multivariable, non-regularized logistic regression analysis. High BP was independently predicted by high EX (OR 1.40, 95% CI 1.16, 1.69, p = 0.001) and clinical depressive symptoms (OR 1.27, 95% CI 1.06, 1.53, p = 0.011) (Supplementary Table S1).Other chronic diseases were also independently predicted by high EX (OR 1.33, 95% CI 1.10, 1.60, p = 0.003) and clinical depressive symptoms (OR 1.46, 95% CI 1.21, 1.75, p < 0.001) (Supplementary Table S2). Chronic lung diseases were independently predicted by low PE (OR 1.47, 95% CI 1.08, 1.99, p = 0.013) (Supplementary Table S3). Finally, skin diseases were independently predicted by clinical depressive symptoms (OR 1.26, 95% CI 1.01, 1.58; p = 0.038) (Supplementary Table S4).

## Discussion

Performing network analysis, we found significant associations of high EX with high BP and a residual category of other chronic somatic diseases, and of low PE with chronic lung diseases, but no associations with high CYN, in respondents to an online survey of job burnout. These associations were adjusted for sociodemographic factors, clinically relevant depressive symptoms, and other somatic diseases and confirmed by logistic regression analysis. More precisely, using a previously proposed cut-off of 3.50 for each burnout dimension^8^, we found high EX to be associated with a 40% and 33% increased relative risk of high BP and other chronic somatic diseases, respectively. Low PE was significantly associated with a 47% increased relative risk of chronic lung diseases. Among other associations, we also observed a significant association of clinical depressive symptoms with high EX on the one hand and with high BP, skin diseases and other chronic somatic diseases on the other. These observations confirm an overlap between depression and burnout^11,12^, but also suggest partially independent associations of depression and burnout with somatic diseases. The overlap between high EX and clinical depressive symptoms on the one hand and their somatic outcomes on the other is perhaps not so surprising, since feelings of exhaustion are part of depressive symptomatology.

Our findings concur with results from some previous studies, but with partly different definitions of burnout as a single score or cut-off scores for high EX, high CYN and low PE than those we used in our study. The advantages and disadvantages of different burnout definitions and measurements have been acknowledged elsewhere^3,28^, and were not part of our study, but must be kept in mind when comparing results between different studies. For instance, high EX, defined by the same cut-off as in our study, was associated with elevated BP in individuals working under highly demanding conditions^10^. Moreover, the total score of the School Burnout Inventory (EX at school, CYN toward the meaning of school, sense of inadequacy at school) was associated with elevated 24-h ambulatory BP in female students^29^. A burnout total score consisting of EX and CYN symptoms was associated with self-reported treatment for hypertension in South African employees^30^. In 5000 civil servants from China, a higher burnout total score (sum of emotional exhaustion, depersonalization, and reduced accomplishment of a 15-item MBI-Human-Services Survey) was associated with a higher risk of chronic diseases, including hypertension, coronary heart disease, stroke, and diabetes^31^.

We did not find an association of burnout dimensions with CVD or cardiovascular risk factors, except high BP. Limited statistical power could explain this, as the prevalence of CVD was low in our sample. The diseases categorized under the residual category “other chronic somatic diseases” are subject to speculation, as this was a residual category which could be selected if participants had a disease form a category which was not specified on the shortlist. For instance, in Turkish physicians, peptic ulcers have previously been associated with high emotional exhaustion, defined by a cut-off of 2.0 on the emotional exhaustion subscale of the 22-item MBI-Human Services Survey^32^. Moreover, as the category of other chronic somatic disease showed a strong relationship with liver disease, and to a lesser extent with pulmonary and skin diseases, one option could be inflammatory bowel disease or systemic diseases with organ manifestations. Unlike a previous study applying a clinical health examination, applying the same cut-off as used in our study^8^, we found the association of low PE with self-reported chronic lung diseases to be significant.

Behavioral and biological mechanisms might be involved in the association between burnout and somatic diseases. For instance, high EX, defined by the same cut-off as in our study, has been associated with low physical activity and heavy alcohol drinking^33^. Also, one explanation for the association between low PE and chronic lung disease could be smoking, which has been shown to be more frequent in employees in the health care sector with low PE than in those with high PE^34^. Greater total frequency and total severity of burnout symptoms, measured with a 23-item early version of the MBI-Human Services Survey, were associated with increased allostatic load, denoting chronic stress-induced biological burden leading to diseases via neuroendocrine, immune and autonomic pathways^35^. However, of the three burnout dimensions, only the emotional exhaustion symptom frequency score was significantly associated with allostatic load, whereas depersonalization and lack of personal accomplishment were not^35^. In contrast, we found no significant association between high CYN and any somatic disease category. The absence of this relationship remains to some extent speculative. For instance, a previous study found an association between depersonalization and alcohol abuse in health care workers^34^. However, since our sample was only 44 years old on average, excessive alcohol consumption may not yet have led to manifest somatic disease in a significant proportion of those with high CYN. Another explanation could be limited statistical power, as only 10.4% of our study participants reported high CYN. The use of network analysis and the large sample size are strengths of our study, which has also its limitations. An investigation of users of a web application, including medical doctors, limits the generalizability of our findings. In addition, we did not validate our results in a second, independent dataset, so the external validity of our results is currently unknown. There was insufficient occupational information regarding job content and environment of study participants; however, it has been argued that the dimensions of the MBI-GS, used in our study, can be applied across all work domains^28^. Self-reported diseases are less accurate than clinical diagnosis. The network model does not allow for unidirect associations. For instance, whereas it is possible that EX has led to high BP through increased sympathetic activity, inflammation, accompanying high BP, could also have triggered sickness behavior including feelings of exhaustion and depressive mood. There could be residual confounding through unmeasured health behaviors. There is no definite consensus on cut-offs that may best define clinically relevant symptoms of burnout and depression with the MBI and POMS, respectively, and results might vary across different psychometric instruments.

Taken together, our network analysis provides evidence that burnout dimensions are significantly associated with an increased risk of somatic diseases. The observed associations were independent of sociodemographic factors and clinically relevant depressive symptoms and more apparent for high EX compared with the other dimensions of the burnout experience.

## Supplementary information

Supplementary Information

## Data Availability

The datasets analysed during the current study are available from the corresponding author on reasonable request.
