# Supplementary material for: Relationship between job burnout and somatic diseases: a network analysis
Source: Sci Rep. 2020 Oct 28;10:18438. doi: 10.1038/s41598-020-75611-7 (PMC7595180; doi:10.1038/s41598-020-75611-7)
Supplement: Supplementary file 1 — Supplementary Information [file 41598_2020_75611_MOESM1_ESM.docx]

**Supplementary Tables 1S-4S**

**Syntax file of analytic code**

**Relationship between job burnout and somatic diseases: a network analysis**

Roland von Känel, M.D.

Mary Princip, Ph.D.

Sarah A. Holzgang, M.Sc.

Walther J. Fuchs, Ph.D.

Marc van Nuffel

Aju P. Pazhenkottil, M.D.

Tobias R. Spiller, M.D.

|  |  |  |  |  |  |  |  |
| --- | --- | --- | --- | --- | --- | --- | --- |
| Predictor | Level |  | High blood pressure, n (%) | |  | Odds Ratio (95% CI, P-Value) | |
|  |  |  | 0 | 1 |  | Univariable | Multivariable |
| Depression | 0 |  | 2015 (85.2) | 350 (14.8) |  | - | - |
|  | 1 |  | 2590 (78.3) | 716 (21.7) |  | 1.59 (1.38-1.83, p<0.001) | 1.27 (1.06-1.53, p=0.011) |
| High exhaustion | 0 |  | 2146 (85.3) | 371 (14.7) |  | - | - |
|  | 1 |  | 2459 (78.0) | 695 (22.0) |  | 1.63 (1.42-1.88, p<0.001) | 1.40 (1.16-1.69, p=0.001) |
| High cynicism | 0 |  | 4153 (81.7) | 931 (18.3) |  | - | - |
|  | 1 |  | 452 (77.0) | 135 (23.0) |  | 1.33 (1.08-1.63, p=0.006) | 1.05 (0.83-1.32, p=0.688) |
| Low personal efficacy | 0 |  | 3114 (83.3) | 625 (16.7) |  | - | - |
|  | 1 |  | 1491 (77.2) | 441 (22.8) |  | 1.47 (1.29-1.69, p<0.001) | 1.06 (0.89-1.26, p=0.542) |
| High Cholesterol | 0 |  | 4061 (85.2) | 703 (14.8) |  | - | - |
|  | 1 |  | 544 (60.0) | 363 (40.0) |  | 3.85 (3.30-4.50, p<0.001) | 2.60 (2.19-3.07, p<0.001) |
| Diabetes Mellitus | 0 |  | 4506 (82.5) | 956 (17.5) |  | - | - |
|  | 1 |  | 99 (47.4) | 110 (52.6) |  | 5.24 (3.96-6.94, p<0.001) | 1.77 (1.23-2.52, p=0.002) |
| Stroke | 0 |  | 4543 (82.1) | 993 (17.9) |  | - | - |
|  | 1 |  | 62 (45.9) | 73 (54.1) |  | 5.39 (3.82-7.63, p<0.001) | 1.13 (0.65-1.90, p=0.657) |
| Myocardial infarction | 0 |  | 4548 (82.3) | 975 (17.7) |  | - | - |
|  | 1 |  | 57 (38.5) | 91 (61.5) |  | 7.45 (5.33-10.49, p<0.001) | 2.17 (1.37-3.40, p=0.001) |
| Heart failure | 0 |  | 4542 (82.2) | 982 (17.8) |  | - | - |
|  | 1 |  | 63 (42.9) | 84 (57.1) |  | 6.17 (4.42-8.64, p<0.001) | 1.68 (1.03-2.68, p=0.032) |
| Lung disease | 0 |  | 4386 (82.3) | 946 (17.7) |  | - | - |
|  | 1 |  | 219 (64.6) | 120 (35.4) |  | 2.54 (2.01-3.20, p<0.001) | 1.11 (0.81-1.51, p=0.494) |
| Liver disease | 0 |  | 4539 (82.3) | 977 (17.7) |  | - | - |
|  | 1 |  | 66 (42.6) | 89 (57.4) |  | 6.26 (4.53-8.70, p<0.001) | 1.83 (1.15-2.86, p=0.009) |
| Renal disease | 0 |  | 4553 (82.2) | 986 (17.8) |  | - | - |
|  | 1 |  | 52 (39.4) | 80 (60.6) |  | 7.10 (4.99-10.19, p<0.001) | 1.99 (1.18-3.30, p=0.008) |
| Skin disease | 0 |  | 4108 (82.1) | 898 (17.9) |  | - | - |
|  | 1 |  | 497 (74.7) | 168 (25.3) |  | 1.55 (1.28-1.86, p<0.001) | 0.95 (0.75-1.19, p=0.638) |
| Other disease | 0 |  | 3814 (82.1) | 829 (17.9) |  | - | - |
|  | 1 |  | 791 (76.9) | 237 (23.1) |  | 1.38 (1.17-1.62, p<0.001) | 0.92 (0.76-1.12, p=0.423) |
| Sex | male |  | 1639 (74.8) | 551 (25.2) |  | - | - |
|  | female |  | 2966 (85.2) | 515 (14.8) |  | 0.52 (0.45-0.59, p<0.001) | 0.54 (0.47-0.63, p<0.001) |
| Age | < 45 |  | 2276 (85.9) | 373 (14.1) |  | - | - |
|  | >45 |  | 2329 (77.1) | 693 (22.9) |  | 1.82 (1.58-2.09, p<0.001) | 1.64 (1.42-1.91, p<0.001) |
| Education Level^1^ | Low |  | 1905 (79.9) | 479 (20.1) |  | - | - |
|  | High |  | 2700 (82.1) | 587 (17.9) |  | 0.86 (0.76-0.99, p=0.034) | 0.90 (0.78-1.04, p=0.166) |

Table 1S. *Predicting high blood pressure*

*Note.*^1^ High defined as university degree, school leaving examination, or vocational school certificate

Overall model: LR-Chi-Square: 570.22, df = 17, p <.001; Nagelkerke R2 = .154, AIC = 4947

|  |  |  |  |  |  |  |  |
| --- | --- | --- | --- | --- | --- | --- | --- |
| Predictor | Level |  | Other somatic disease, n (%) | |  | Odds Ratio (95% CI, P-Value) | |
|  |  |  | 0 | 1 |  | Univariable | Multivariable |
| Depression | 0 |  | 1909 (86.9) | 289 (13.1) |  | - | - |
|  | 1 |  | 2734 (78.7) | 739 (21.3) |  | 1.79 (1.54-2.07, p<0.001) | 1.46 (1.21-1.75, p<0.001) |
| High exhaustion | 0 |  | 2161 (85.9) | 356 (14.1) |  | - | - |
|  | 1 |  | 2482 (78.7) | 672 (21.3) |  | 1.64 (1.43-1.89, p<0.001) | 1.33 (1.10-1.60, p=0.003) |
| High cynicism | 0 |  | 4174 (82.1) | 910 (17.9) |  | - | - |
|  | 1 |  | 469 (79.9) | 118 (20.1) |  | 1.15 (0.93-1.42, p=0.190) | 0.86 (0.67-1.08, p=0.204) |
| Low personal efficacy | 0 |  | 3109 (83.2) | 630 (16.8) |  | - | - |
|  | 1 |  | 1534 (79.4) | 398 (20.6) |  | 1.28 (1.11-1.47, p=0.001) | 0.89 (0.75-1.07, p=0.211) |
| High Cholesterol | 0 |  | 3984 (83.6) | 780 (16.4) |  | - | - |
|  | 1 |  | 659 (72.7) | 248 (27.3) |  | 1.92 (1.63-2.26, p<0.001) | 1.39 (1.14-1.68, p=0.001) |
| Diabetes Mellitus | 0 |  | 4530 (82.9) | 932 (17.1) |  | - | - |
|  | 1 |  | 113 (54.1) | 96 (45.9) |  | 4.13 (3.11-5.47, p<0.001) | 1.50 (1.01-2.19, p=0.040) |
| Stroke | 0 |  | 4591 (82.9) | 945 (17.1) |  | - | - |
|  | 1 |  | 52 (38.5) | 83 (61.5) |  | 7.75 (5.46-11.10, p<0.001) | 2.32 (1.41-3.76, p=0.001) |
| Myocardial infarction | 0 |  | 4568 (82.7) | 955 (17.3) |  | - | - |
|  | 1 |  | 75 (50.7) | 73 (49.3) |  | 4.66 (3.35-6.48, p<0.001) | 0.79 (0.44-1.34, p=0.389) |
| Heart failure | 0 |  | 4577 (82.9) | 947 (17.1) |  | - | - |
|  | 1 |  | 66 (44.9) | 81 (55.1) |  | 5.93 (4.26-8.29, p<0.001) | 1.45 (0.87-2.36, p=0.143) |
| Lung disease | 0 |  | 4454 (83.5) | 878 (16.5) |  | - | - |
|  | 1 |  | 189 (55.8) | 150 (44.2) |  | 4.03 (3.21-5.05, p<0.001) | 2.05 (1.55-2.69, p<0.001) |
| Liver disease | 0 |  | 4589 (83.2) | 927 (16.8) |  | - | - |
|  | 1 |  | 54 (34.8) | 101 (65.2) |  | 9.26 (6.63-13.06, p<0.001) | 3.44 (2.22-5.32, p<0.001) |
| Renal disease | 0 |  | 4592 (82.9) | 947 (17.1) |  | - | - |
|  | 1 |  | 51 (38.6) | 81 (61.4) |  | 7.70 (5.41-11.07, p<0.001) | 1.37 (0.80-2.31, p=0.242) |
| Skin disease | 0 |  | 4210 (84.1) | 796 (15.9) |  | - | - |
|  | 1 |  | 433 (65.1) | 232 (34.9) |  | 2.83 (2.37-3.38, p<0.001) | 1.89 (1.54-2.31, p<0.001) |
| High blood pressure | 0 |  | 3814 (82.8) | 791 (17.2) |  | - | - |
|  | 1 |  | 829 (77.8) | 237 (22.2) |  | 1.38 (1.17-1.62, p<0.001) | 0.92 (0.76-1.12, p=0.407) |
| Sex | male |  | 1903 (86.9) | 287 (13.1) |  | - | - |
|  | female |  | 2740 (78.7) | 741 (21.3) |  | 1.79 (1.55-2.08, p<0.001) | 1.90 (1.62-2.23, p<0.001) |
| Age | < 45 |  | 2236 (84.4) | 413 (15.6) |  | - | - |
|  | >45 |  | 2407 (79.6) | 615 (20.4) |  | 1.38 (1.21-1.59, p<0.001) | 1.44 (1.24-1.67, p<0.001) |
| Education Level^1^ | Low |  | 1868 (78.4) | 516 (21.6) |  | - | - |
|  | High |  | 2775 (84.4) | 512 (15.6) |  | 0.67 (0.58-0.76, p<0.001) | 0.73 (0.63-0.85, p<0.001) |

Table 2S. *Predicting other somatic disease*

*Note.*^1^ High defined as university degree, school leaving examination, or vocational school certificate

Overall model: LR-Chi-Square: 497.02, df = 17, p <.001; Nagelkerke R2 = .137, AIC = 4907.4

|  |  |  |  |  |  |  |  |
| --- | --- | --- | --- | --- | --- | --- | --- |
| Predictor | Level |  | Lung disease, n (%) | |  | Odds Ratio (95% CI, P-Value) | |
|  |  |  | 0 | 1 |  | Univariable | Multivariable |
| Depression | 0 |  | 2103 (95.7) | 95 (4.3) |  | - | - |
|  | 1 |  | 3229 (93.0) | 244 (7.0) |  | 1.67 (1.32-2.14, p<0.001) | 1.14 (0.83-1.59, p=0.419) |
| High exhaustion | 0 |  | 2398 (95.3) | 119 (4.7) |  | - | - |
|  | 1 |  | 2934 (93.0) | 220 (7.0) |  | 1.51 (1.20-1.91, p<0.001) | 1.00 (0.72-1.39, p=1.000) |
| High cynicism | 0 |  | 4793 (94.3) | 291 (5.7) |  | - | - |
|  | 1 |  | 539 (91.8) | 48 (8.2) |  | 1.47 (1.06-2.00, p=0.018) | 0.89 (0.59-1.31, p=0.571) |
| Low personal efficacy | 0 |  | 3556 (95.1) | 183 (4.9) |  | - | - |
|  | 1 |  | 1776 (91.9) | 156 (8.1) |  | 1.71 (1.37-2.13, p<0.001) | 1.47 (1.08-1.99, p=0.013) |
| High Cholesterol | 0 |  | 4550 (95.5) | 214 (4.5) |  | - | - |
|  | 1 |  | 782 (86.2) | 125 (13.8) |  | 3.40 (2.69-4.28, p<0.001) | 1.60 (1.17-2.17, p=0.003) |
| Diabetes Mellitus | 0 |  | 5195 (95.1) | 267 (4.9) |  | - | - |
|  | 1 |  | 137 (65.6) | 72 (34.4) |  | 10.23 (7.47-13.91, p<0.001) | 1.41 (0.76-2.44, p=0.252) |
| Stroke | 0 |  | 5263 (95.1) | 273 (4.9) |  | - | - |
|  | 1 |  | 69 (51.1) | 66 (48.9) |  | 18.44 (12.87-26.41, p<0.001) | 1.20 (0.52-2.51, p=0.657) |
| Myocardial infarction | 0 |  | 5256 (95.2) | 267 (4.8) |  | - | - |
|  | 1 |  | 76 (51.4) | 72 (48.6) |  | 18.65 (13.20-26.34, p<0.001) | 3.00 (1.58-5.41, p<0.001) |
| Heart failure | 0 |  | 5259 (95.2) | 265 (4.8) |  | - | - |
|  | 1 |  | 73 (49.7) | 74 (50.3) |  | 20.12 (14.23-28.46, p<0.001) | 2.53 (1.32-4.58, p=0.004) |
| Other disease | 0 |  | 4454 (95.9) | 189 (4.1) |  | - | - |
|  | 1 |  | 878 (85.4) | 150 (14.6) |  | 4.03 (3.21-5.05, p<0.001) | 2.00 (1.51-2.63, p<0.001) |
| Liver disease | 0 |  | 5252 (95.2) | 264 (4.8) |  | - | - |
|  | 1 |  | 80 (51.6) | 75 (48.4) |  | 18.65 (13.29-26.17, p<0.001) | 1.75 (0.91-3.17, p=0.078) |
| Renal disease | 0 |  | 5279 (95.3) | 260 (4.7) |  | - | - |
|  | 1 |  | 53 (40.2) | 79 (59.8) |  | 30.26 (20.98-44.00, p<0.001) | 5.42 (2.98-9.51, p<0.001) |
| Skin disease | 0 |  | 4790 (95.7) | 216 (4.3) |  | - | - |
|  | 1 |  | 542 (81.5) | 123 (18.5) |  | 5.03 (3.96-6.38, p<0.001) | 2.12 (1.55-2.87, p<0.001) |
| High blood pressure | 0 |  | 4386 (95.2) | 219 (4.8) |  | - | - |
|  | 1 |  | 946 (88.7) | 120 (11.3) |  | 2.54 (2.01-3.20, p<0.001) | 1.11 (0.80-1.51, p=0.523) |
| Sex | male |  | 2067 (94.4) | 123 (5.6) |  | - | - |
|  | female |  | 3265 (93.8) | 216 (6.2) |  | 1.11 (0.89-1.40, p=0.363) | 1.14 (0.87-1.48, p=0.348) |
| Age | < 45 |  | 2483 (93.7) | 166 (6.3) |  | - | - |
|  | >45 |  | 2849 (94.3) | 173 (5.7) |  | 0.91 (0.73-1.13, p=0.391) | 0.84 (0.65-1.08, p=0.180) |
| Education Level^1^ | Low |  | 2231 (93.6) | 153 (6.4) |  | - | - |
|  | High |  | 3101 (94.3) | 186 (5.7) |  | 0.87 (0.70-1.09, p=0.234) | 0.97 (0.75-1.25, p=0.814) |

Table 3S. *Predicting lung disease*

*Note.*^1^ High defined as university degree, school leaving examination, or vocational school certificate

Overall model: LR-Chi-Square: 483.01, df = 17, p <.001; Nagelkerke R2 = .224, AIC = 2120.5

|  |  |  |  |  |  |  |  |
| --- | --- | --- | --- | --- | --- | --- | --- |
| Predictor | Level |  | Skin disease pressure, n (%) | |  | Odds Ratio (95% CI, P-Value) | |
|  |  |  | 0 | 1 |  | Univariable | Multivariable |
| Depression | 0 |  | 1993 (90.7) | 205 (9.3) |  | - | - |
|  | 1 |  | 3013 (86.8) | 460 (13.2) |  | 1.48 (1.25-1.77, p<0.001) | 1.26 (1.01-1.58, p=0.038) |
| High exhaustion | 0 |  | 2268 (90.1) | 249 (9.9) |  | - | - |
|  | 1 |  | 2738 (86.8) | 416 (13.2) |  | 1.38 (1.17-1.64, p<0.001) | 1.15 (0.92-1.45, p=0.209) |
| High cynicism | 0 |  | 4492 (88.4) | 592 (11.6) |  | - | - |
|  | 1 |  | 514 (87.6) | 73 (12.4) |  | 1.08 (0.83-1.39, p=0.572) | 0.78 (0.57-1.04, p=0.096) |
| Low personal efficacy | 0 |  | 3332 (89.1) | 407 (10.9) |  | - | - |
|  | 1 |  | 1674 (86.6) | 258 (13.4) |  | 1.26 (1.07-1.49, p=0.006) | 1.00 (0.81-1.23, p=0.975) |
| High Cholesterol | 0 |  | 4265 (89.5) | 499 (10.5) |  | - | - |
|  | 1 |  | 741 (81.7) | 166 (18.3) |  | 1.91 (1.58-2.32, p<0.001) | 1.19 (0.94-1.50, p=0.152) |
| Diabetes Mellitus | 0 |  | 4878 (89.3) | 584 (10.7) |  | - | - |
|  | 1 |  | 128 (61.2) | 81 (38.8) |  | 5.29 (3.94-7.06, p<0.001) | 1.45 (0.92-2.23, p=0.097) |
| Stroke | 0 |  | 4942 (89.3) | 594 (10.7) |  | - | - |
|  | 1 |  | 64 (47.4) | 71 (52.6) |  | 9.23 (6.52-13.10, p<0.001) | 1.69 (0.94-2.90, p=0.070) |
| Myocardial infarction | 0 |  | 4925 (89.2) | 598 (10.8) |  | - | - |
|  | 1 |  | 81 (54.7) | 67 (45.3) |  | 6.81 (4.86-9.51, p<0.001) | 0.96 (0.51-1.71, p=0.894) |
| Heart failure | 0 |  | 4935 (89.3) | 589 (10.7) |  | - | - |
|  | 1 |  | 71 (48.3) | 76 (51.7) |  | 8.97 (6.42-12.55, p<0.001) | 1.93 (1.11-3.21, p=0.015) |
| Other disease | 0 |  | 4210 (90.7) | 433 (9.3) |  | - | - |
|  | 1 |  | 796 (77.4) | 232 (22.6) |  | 2.83 (2.37-3.38, p<0.001) | 1.88 (1.53-2.29, p<0.001) |
| Liver disease | 0 |  | 4938 (89.5) | 578 (10.5) |  | - | - |
|  | 1 |  | 68 (43.9) | 87 (56.1) |  | 10.93 (7.88-15.22, p<0.001) | 2.85 (1.77-4.50, p<0.001) |
| Renal disease | 0 |  | 4950 (89.4) | 589 (10.6) |  | - | - |
|  | 1 |  | 56 (42.4) | 76 (57.6) |  | 11.41 (8.01-16.34, p<0.001) | 1.95 (1.10-3.35, p=0.018) |
| Lung disease | 0 |  | 4790 (89.8) | 542 (10.2) |  | - | - |
|  | 1 |  | 216 (63.7) | 123 (36.3) |  | 5.03 (3.96-6.38, p<0.001) | 2.20 (1.62-2.96, p<0.001) |
| High blood pressure | 0 |  | 4108 (89.2) | 497 (10.8) |  | - | - |
|  | 1 |  | 898 (84.2) | 168 (15.8) |  | 1.55 (1.28-1.86, p<0.001) | 0.97 (0.76-1.21, p=0.761) |
| Sex | male |  | 1952 (89.1) | 238 (10.9) |  | - | - |
|  | female |  | 3054 (87.7) | 427 (12.3) |  | 1.15 (0.97-1.36, p=0.111) | 1.12 (0.93-1.34, p=0.235) |
| Age | < 45 |  | 2326 (87.8) | 323 (12.2) |  | - | - |
|  | >45 |  | 2680 (88.7) | 342 (11.3) |  | 0.92 (0.78-1.08, p=0.306) | 0.88 (0.74-1.05, p=0.153) |
| Education Level^1^ | Low |  | 2113 (88.6) | 271 (11.4) |  | - | - |
|  | High |  | 2893 (88.0) | 394 (12.0) |  | 1.06 (0.90-1.25, p=0.474) | 1.19 (1.00-1.42, p=0.057) |

Table 4S. *Predicting skin disease*

*Note.*^1^ High defined as university degree, school leaving examination, or vocational school certificate

Overall model: LR-Chi-Square: 362.12, df = 17, p <.001; Nagelkerke R2 = .120, AIC = 3773.3

##############################################################################

# #

# Network analysis of the relationship between #

# burnout and somatic diseases #

# #

# von Känel et al. Sci Rep 2020 #

# #

# Code Version 4.0 (04.08.2020) #

# #

#----------------------------------------------------------------------------#

# #

# Questions concerning this code: tobias.r.spiller@gmail.com #

# #

##############################################################################

###### Table of Contents #####################################################

#----- 1. Load libraries ----------------------------------------------------#

#----- 2. Import and prepare data -------------------------------------------#

#----- 3. Descriptive -------------------------------------------------------#

#----- 4. Network Analysis ------------------------------------------------#

#----- 5. Logistic Regression - nominal -------------------------------------#

#----- 6. Logistic Regression - continuous ----------------------------------#

#----- 7. Session info ------------------------------------------------------#

## Notes ##

# Name of datasets

# bp_export_09092019.csv = raw data

# cleaned.master.df = dataset with all variables

# data1 = dataset with all variables needed to characterize the sample and for analysis

# data2 = fully cleaned and prepared dataset

# data3 = selected variables for NW & Logistic regression

# data3_NW = selected variables for NW

###### 1. Load Libraries #####################################################

# Data handling

if(!require("tidyverse")) install.packages("tidyverse")

if(!require("readxl")) install.packages("readxl")

if(!require("psych")) install.packages("psych")

if(!require("yarrr")) install.packages("yarrr")

# Regression

if(!require("rms")) install.packages("rms")

if(!require("car")) install.packages("car")

if(!require("finalfit")) install.packages("finalfit")

# Network specific

if(!require("qgraph")) install.packages("qgraph")

if(!require("bootnet")) install.packages("bootnet")

###### 2. Import and prepare data ############################################

#### Import data

# protector.all <- read.csv("~/Desktop/MBI_Somat_Dg/bp_export_09092019.csv")

#### Prepare dataset

### Clean dataset

# Select numeric values (every second row with some adjustments)

protector_values <- protector.all[,seq(1, ncol(protector.all), 2)]

protector_values<- protector_values[,(1:86)] #the 86th coloum does not have an index an values, but only values

protector_values$ID <- seq.int(nrow(protector_values)) # add ID

protector_values2 <- protector.all[,seq(2, ncol(protector.all), 2)]

protector_values2<- protector_values2[,(86:99)]

protector_values2$ID <- seq.int(nrow(protector_values2)) # add ID

#Merge

cleaned.master.df <- merge(protector_values,protector_values2, by= "ID")

#Extract column names

colnames.protect <- colnames(cleaned.master.df)

colnames.protect <- as.data.frame(colnames.protect)

#Save names as .csv

write.csv(colnames.protect, file = "variables.csv", row.names = TRUE)

#Rename colums

names.df <- read_excel("names.xlsx") # Cleaned names list

names.df <- t(names.df)

colnames(names.df) = names.df[3, ] # the second row will be the header

colnames(cleaned.master.df) <- colnames(names.df)

### Select variables of importance and descirptive values

selected_variables_df <- dplyr::select(cleaned.master.df, "user", "age", "sex", "edu", "fam", (mbi_1:mbi_16),

"hypertension", "cholest", "diabetes", "stroke", "myocardinfarct",

"congest HF", "pulmo", "liver", "nephro", "skin", "other_disorder", (POMS_1:POMS_35),"doc_clin", "doc_other")

# Select 18-70 years old

selected_variables_df_age <- subset(selected_variables_df, age < 71)

# Select only enrty one per user

selected_variables_df_age$duplicate <- duplicated(selected_variables_df_age$user)

selected_variables_df_age_dup <- selected_variables_df_age %>%

filter(duplicate == FALSE)

data1 <- selected_variables_df_age_dup

## Caluclate scores for MBI and POMS (incl. the subscales)

# POMS: Depression >= 14.4, (adjusted for 14 items from POMS =16 (with 15 items) ~ BDI-II = 20, see:

# Griffith, N. M., Szaflarski, J. P., Szaflarski, M., Kent, G. P., Schefft, B. K., Howe, S. R., & Privitera, M. D. (2005).

# Measuring depressive symptoms among treatment-resistant seizure disorder patients: POMS Depression scale as an

# alternative to the BDI-II. Epilepsy & Behavior, 7(2), 266-272.

# MBI

data1.extended <- mutate(data1,

MBI_EE = (mbi_1+ mbi_2+ mbi_3+ mbi_4+ mbi_6)/5,

MBI_DP = (mbi_5+ mbi_7+ mbi_10+ mbi_11+ mbi_16)/5,

MBI_PA = (mbi_8+ mbi_9+ mbi_12+ mbi_13+ mbi_14 + mbi_15)/6)

data1.extended <- mutate(data1.extended,

Burnout_severity = ((0.4*MBI_EE) + (0.3*MBI_DP) + (0.3*MBI_PA)))

# POMS

data1.extended <- mutate(data1.extended,

Niedergeschlagen = (POMS_3 + POMS_5 + POMS_7 + POMS_11 + POMS_13 +

POMS_14 + POMS_18 + POMS_19 + POMS_21+ POMS_23 +

POMS_24 + POMS_26 + POMS_32 + POMS_33),

Niedergeschlagen_Mean = Niedergeschlagen/14,

Müdigkeit = (POMS_2 + POMS_6 + POMS_16 + POMS_22 + POMS_25 + POMS_27 + POMS_35),

Missmut = (POMS_1 + POMS_9 + POMS_10 + POMS_15 + POMS_17 + POMS_29 + POMS_31))

# Binarize data (Burnout yes/no, Burnout subscales: severe yes/no ; medium yes/no, Depression yes/no, )

data1.extended <- mutate(data1.extended,

EE_3_5 = case_when(MBI_EE > 3.5 ~1, MBI_EE <= 3.5 ~0),

DP_3_5 = case_when(MBI_DP > 3.5 ~1, MBI_DP <= 3.5 ~0),

PA_3_5 = case_when(MBI_PA > 3.5 ~1, MBI_PA <= 3.5 ~0),

Burnout_3_5 = case_when(Burnout_severity > 3.5 ~1, Burnout_severity <= 3.5 ~0),

Dep = case_when(Niedergeschlagen >= 14.0 ~1, Niedergeschlagen < 14.0 ~ 0), ## VORHER: 13.067

Age_bin = case_when(age >= 45 ~1, age < 45 ~ 0),

Edu_bin = case_when(edu >= 3 ~1, edu < 3 ~ 0))

## INF : NA

#As factors and add labels

# Select only cases with complete values for network (but missing in descriptives is fine)

data2 <- data1.extended%>%

drop_na(Burnout_severity, hypertension, cholest, diabetes, stroke, myocardinfarct,

"congest HF", pulmo, liver, nephro, skin, other_disorder, (mbi_1:mbi_16), age, sex, edu, Dep)

data2$sex <- factor(data2$sex,labels = c("male", "female"))

data2$edu <- factor(data2$edu, labels = c("1 = Obligatorische Schulzeit (od. weniger), Anlehre",

"2 = Berufslehre, Handelsschule, Diplommittelschule",

"3 = Gymnasiale Matura, Berufsmatura",

"4 = Fachhochschule, Technikum, Universitätsabschluss"))

data2$fam <- factor(data2$fam, labels = c("lebt alleine", "Lebt mit jemandem zusammen"))

data2$Age_bin <- factor(data2$Age_bin, labels = c("< 45", ">45"))

data2$Edu_bin <- factor(data2$Edu_bin, labels = c("max Lehre", "mind BMS"))

#As facors without labels

cols <- c("EE_3_5", "DP_3_5", "PA_3_5", "Dep", "Burnout_3_5",

"hypertension", "cholest", "diabetes", "stroke", "myocardinfarct",

"congest HF", "pulmo", "liver", "nephro", "skin", "other_disorder", "doc_clin")

data2[cols] <- lapply(data2[cols], factor)

# Save data as .csv

# write.csv2(data2, "clean_prep_mbi_somat_dg.csv")

# Extract relevant variables for NW and logistic regression

data3 <- data2 %>%

select("Dep","EE_3_5", "DP_3_5", "PA_3_5","hypertension", "cholest", "diabetes", "stroke", "myocardinfarct",

"congest HF", "pulmo", "liver", "nephro", "skin", "other_disorder", "MBI_EE", "MBI_PA", "MBI_DP", "Niedergeschlagen_Mean",

"age", "sex", "edu", "Age_bin", "Edu_bin", "user", "doc_clin", "doc_other")

# Save data as .csv

# write.csv2(data3, "mbi_somat_dg.csv")

###### 3. Descriptives #######################################################

summary(data3)

psych::describe(data2)

###### 4. Network Analysis ###################################################

## Prepare data

# Rename variables

colnames(data3) <- c("DEP", "EX", "CY", "PE","HYP", "CHOL", "DM", "STRO", "MI",

"HF", "PULM", "LIV", "NEPH", "SKIN", "OTHR", "EE_CONT", "PA_CONT", "DP_CONT", "NIEDER_Mean", "AGE", "SEX", "EDU_ord", "AGE_45", "EDU","user", "doc_clin", "doc_other")

## Network Model

# exclude continous variables

set.seed(241)

data3_NW <- data3 %>%

select("DEP", "EX", "CY", "PE","HYP", "CHOL", "DM", "STRO", "MI",

"HF", "PULM", "LIV", "NEPH", "SKIN", "OTHR", "SEX", "AGE_45", "EDU")

data3_NW_num <- data.matrix(data3_NW)

## Estimate Network

network3_5 <- estimateNetwork(data3_NW_num, default = "IsingFit")

Colors <- yarrr::piratepal(palette = "southpark")

group1 = c("1", "1", "1", "1", "2","2","2","2","2","2","2","2","2","2","2",

"3","3","3")

# Plot Network

pdf("NW_Burnout_Somat_dg.pdf")

plot(network3_5, layout = "circle",groups=group1, color=Colors, legend = FALSE)

dev.off()

# as.tiff

tiff("NW_Burnout_Somat_dg.tiff", units="in", width=5, height=5, res=400)

plot(network3_5, layout = "circle",groups=group1, color=Colors, legend = FALSE)

dev.off()

setEPS()

postscript("NW_Burnout_Somat_dg.eps")

plot(network3_5, layout = "circle",groups=group1, color=Colors, legend = FALSE)

dev.off()

# alternative estimation

# network3_5 <- bootnet_IsingFit(data3_NW_num, tuning = 0.5, missing = "stop", verbose = TRUE,

# rule = "OR", principalDirection = FALSE)

# Adjunct Matrix

# To get OR: x= coefficients, e^x = OR

network3_5$results$weiadj

coeff.df <- as.data.frame(network3_5$results$weiadj)

write.csv2(coeff.df, "Coefficients_Network.csv")

# Robustness

### Estimate Stability and Accuracy ###

# Bootstrap

boot1a <- bootnet(network3_5, nBoots = 1000, nCores = 6)

boot1b<- bootnet(network3_5, nBoots = 1000, type = "case", nCores = 6)

## Plot Edge Weight Confidence Intervals ##

pdf("bootnet_Edge_weights_NW_3_5.pdf")

plot(boot1a, order = "sample")

dev.off()

### Conduct and Plot Edge Weights Difference test ###

pdf("bootnet_Edge_difference_NW_3_5.pdf")

plot(boot1a, "edge", plot = "difference", onlyNonZero = TRUE, order = "sample")

dev.off()

## Save output ##

# save(boot1a, file = "boot1a.Rdata")

# save(boot1b, file = "boot1b.Rdata")

###### 5. Logistic Regression - nominal #######################################

### Variables of interest identified with network

### Hypertension

# Full model

glm.HYP_3.5 <- glm(HYP ~ DEP + EX + CY + PE + CHOL + DM + STRO + MI + HF + PULM + LIV + NEPH + SKIN + OTHR + SEX + AGE_45 + EDU, data = data3, family = binomial)

summary(glm.HYP_3.5)

#CI

confint(glm.HYP_3.5)

# Multicollinearity

car::vif(glm.HYP_3.5)

#Export Table

explanatory_HYP = c("DEP", "EX", "CY", "PE", "CHOL", "DM", "STRO", "MI",

"HF", "PULM", "LIV", "NEPH", "SKIN", "OTHR", "SEX", "AGE_45", "EDU")

dependent_HYP = "HYP"

Table_HYP <- data3_NW %>%

finalfit(dependent, explanatory, metrics=TRUE)

# Null model

glm.HYP_3.5_null <- glm(HYP ~ 1, data = data3, family = binomial)

#p-value for McF. pseudo R2

glm.HYP_3.5_LL.null <- glm.HYP_3.5$null.deviance/-2

glm.HYP_3.5_LL.proposed <- glm.HYP_3.5$deviance/-2

## McF. pseudo R2 diff way to calculate

(glm.HYP_3.5_LL.null-glm.HYP_3.5_LL.proposed) / glm.HYP_3.5_LL.null # Pseudo R2 = 0.10

#p-value for R2

1- pchisq(2*(glm.HYP_3.5_LL.proposed-glm.HYP_3.5_LL.null), df=(length(glm.HYP_3.5$coefficients)-1)) #sig.

## Nagelkerke R2

# Different package, easier to get Nagelkere

HYP_Nagel <- lrm(HYP ~ DEP + EX + CY + PE + CHOL + DM + STRO + MI + HF + PULM + LIV + NEPH + SKIN + OTHR + SEX + AGE_45 + EDU, data = data3)

print(HYP_Nagel) # Pseudo R2 Nagelkerke = 0.155

### Other disorders

# Full model

glm.OTHR_3.5 <- glm(OTHR ~ DEP + EX + CY + PE + HYP + CHOL + DM + STRO + MI + HF + PULM + LIV + NEPH + SKIN + SEX + AGE_45 + EDU, data = data3, family = binomial)

summary(glm.OTHR_3.5)

#CI

confint(glm.OTHR_3.5)

# Multicollinearity

car::vif(glm.OTHR_3.5)

# McF. pseudo R2

glm.OTHR_3.5_LL.null <- glm.OTHR_3.5$null.deviance/-2

glm.OTHR_3.5_LL.proposed <- glm.OTHR_3.5$deviance/-2

(glm.OTHR_3.5_LL.null-glm.OTHR_3.5_LL.proposed) / glm.OTHR_3.5_LL.null # 0.09

#p-value for R2

1- pchisq(2*(glm.OTHR_3.5_LL.proposed-glm.OTHR_3.5_LL.null), df=(length(glm.OTHR_3.5$coefficients)-1)) #sig.

## Nagelkerke R2

# Different package, easier to get Nagelkere

OTHR_Nagel <- lrm(OTHR ~ DEP + EX + CY + PE + CHOL + DM + STRO + MI + HF + PULM + LIV + NEPH + SKIN + HYP + SEX + AGE_45 + EDU, data = data3)

print(OTHR_Nagel) # Pseudo R2 Nagelkerke = 0.136

#Export Table

explanatory_OTHR = c("DEP", "EX", "CY", "PE", "CHOL", "DM", "STRO", "MI",

"HF", "PULM", "LIV", "NEPH", "SKIN", "HYP", "SEX", "AGE_45", "EDU")

dependent_OTHR = "OTHR"

Table_OTHR <- data3_NW %>%

finalfit(dependent, explanatory, metrics=TRUE)

### Pulm. Disorders

# Full model

glm.PULM_3.5 <- glm(PULM ~ DEP + EX + CY + PE + CHOL + DM + STRO + MI + HF + HYP + LIV + NEPH + SKIN + OTHR + SEX + AGE_45 + EDU, data = data3, family = binomial)

summary(glm.PULM_3.5)

#CI

confint(glm.PULM_3.5)

# Multicollinearity

car::vif(glm.PULM_3.5)

# Null model

glm.PULM_3.5_null <- glm(PULM ~ 1, data = data3, family = binomial)

#p-value for McF. pseudo R2

glm.PULM_3.5_LL.null <- glm.PULM_3.5$null.deviance/-2

glm.PULM_3.5_LL.proposed <- glm.PULM_3.5$deviance/-2

## McF. pseudo R2 diff way to calculate

(glm.PULM_3.5_LL.null-glm.PULM_3.5_LL.proposed) / glm.PULM_3.5_LL.null # Pseudo R2 = 0.19

#p-value for R2

1- pchisq(2*(glm.PULM_3.5_LL.proposed-glm.PULM_3.5_LL.null), df=(length(glm.PULM_3.5$coefficients)-1)) #sig.

## Nagelkerke R2

# Different package, easier to get Nagelkere

PULM_Nagel <- lrm(PULM ~ DEP + EX + CY + PE + CHOL + DM + STRO + MI + HF + HYP + LIV + NEPH + SKIN + OTHR + SEX + AGE_45 + EDU, data = data3)

print(PULM_Nagel) # Pseudo R2 Nagelkerke = 0.224

explanatory_PULM = c("DEP", "EX", "CY", "PE", "CHOL", "DM", "STRO", "MI",

"HF", "OTHR", "LIV", "NEPH", "SKIN", "HYP", "SEX", "AGE_45", "EDU")

dependent_PULM = "PULM"

Table_PULM <- data3_NW %>%

finalfit(dependent, explanatory, metrics=TRUE)

### Skin disorders

glm.SKIN_3.5 <- glm(SKIN ~ DEP + EX + CY + PE + HYP + CHOL + DM + STRO + MI + HF + PULM + LIV + NEPH + OTHR + SEX + AGE_45 + EDU, data = data3, family = binomial)

summary(glm.SKIN_3.5)

#CI

confint(glm.SKIN_3.5)

# Multicollinearity

car::vif(glm.SKIN_3.5)

## Nagelkerke R2

SKIN_Nagel <- lrm(SKIN ~ DEP + EX + CY + PE + CHOL + DM + STRO + MI + HF + HYP + LIV + NEPH + PULM + OTHR + SEX + AGE_45 + EDU, data = data3)

print(SKIN_Nagel) # Pseudo R2 Nagelkerke = 0.224

explanatory_SKIN = c("DEP", "EX", "CY", "PE", "CHOL", "DM", "STRO", "MI",

"HF", "OTHR", "LIV", "NEPH", "PULM", "HYP", "SEX", "AGE_45", "EDU")

dependent_SKIN = "SKIN"

Table_SKIN <- data3_NW %>%

finalfit(dependent, explanatory, metrics=TRUE)

###### 6. Logistic Regression - continuous ##############################################

# Burnout continuous: Variables of interest identified with Network

# HYP

glm.HYP_cont <- glm(HYP ~ NIEDER_Mean + EE_CONT + DP_CONT + PA_CONT + CHOL + DM + STRO + MI + HF + PULM + LIV + NEPH + SKIN + OTHR + SEX + AGE + EDU, data = data3, family = binomial)

summary(glm.HYP_cont)

# Multicollinearity

car::vif(glm.HYP_cont)

# OTHR

glm.OTHR_cont <- glm(OTHR ~ NIEDER_Mean + EE_CONT + DP_CONT + PA_CONT + HYP + CHOL + DM + STRO + MI + HF + PULM + LIV + NEPH + SKIN + SEX + AGE + EDU, data = data3, family = binomial)

summary(glm.OTHR_cont)

# Multicollinearity

car::vif(glm.OTHR_cont)

# PULM

glm.PULM_cont <- glm(PULM ~ NIEDER_Mean + EE_CONT + DP_CONT + PA_CONT + HYP + CHOL + DM + STRO + MI + HF + LIV + NEPH + SKIN + OTHR + SEX + AGE + EDU, data = data3, family = binomial)

summary(glm.PULM_cont)

# Multicollinearity

car::vif(glm.PULM_cont)

# SKIN

glm.SKIN_cont <- glm(SKIN ~ NIEDER_Mean + EE_CONT + DP_CONT + PA_CONT + HYP + CHOL + DM + STRO + MI + HF + PULM + LIV + NEPH + OTHR + SEX + AGE + EDU, data = data3, family = binomial)

summary(glm.PULM_cont)

###### 7. Session info #######################################################

sessionInfo()

# R version 3.6.1 (2019-07-05)

# Platform: x86_64-apple-darwin15.6.0 (64-bit)

# Running under: macOS Catalina 10.15.1

#

# Matrix products: default

# BLAS: /System/Library/Frameworks/Accelerate.framework/Versions/A/Frameworks/vecLib.framework/Versions/A/libBLAS.dylib

# LAPACK: /Library/Frameworks/R.framework/Versions/3.6/Resources/lib/libRlapack.dylib

#

# locale:

# [1] de_CH.UTF-8/de_CH.UTF-8/de_CH.UTF-8/C/de_CH.UTF-8/de_CH.UTF-8

#

# attached base packages:

# [1] stats graphics grDevices utils datasets methods base

#

# other attached packages:

# [1] mgm_1.2-7 bootnet_1.2.4 qgraph_1.6.3 rms_5.1-3.1 SparseM_1.77 Hmisc_4.2-0 Formula_1.2-3 survival_2.44-1.1 lattice_0.20-38 psych_1.8.12 readxl_1.3.1

# [12] forcats_0.4.0 stringr_1.4.0 dplyr_0.8.3 purrr_0.3.2 readr_1.3.1 tidyr_1.0.0 tibble_2.1.3 ggplot2_3.2.1 tidyverse_1.2.1

#

# loaded via a namespace (and not attached):

# [1] backports_1.1.5 BDgraph_2.61 plyr_1.8.4 igraph_1.2.4.1 lazyeval_0.2.2 splines_3.6.1 TH.data_1.0-10 candisc_0.8-0 digest_0.6.21

# [10] foreach_1.4.7 htmltools_0.4.0 matrixcalc_1.0-3 gdata_2.18.0 magrittr_1.5 checkmate_1.9.4 cluster_2.1.0 doParallel_1.0.15 etm_1.0.5

# [19] openxlsx_4.1.0.1 longitudinal_1.1.12 modelr_0.1.5 wordcloud_2.6 R.utils_2.9.0 sandwich_2.5-1 jpeg_0.1-8 colorspace_1.4-1 rvest_0.3.4

# [28] mitools_2.4 haven_2.1.1 pan_1.6 xfun_0.10 jsonlite_1.6 crayon_1.3.4 networktools_1.2.1 lme4_1.1-21 zeallot_0.1.0

# [37] zoo_1.8-6 iterators_1.0.12 glue_1.3.1 relaimpo_2.2-3 gtable_0.3.0 nnls_1.4 NetworkToolbox_1.3.1 MatrixModels_0.4-1 car_3.0-3

# [46] weights_1.0 ggm_2.3 jomo_2.6-9 abind_1.4-5 scales_1.0.0 mvtnorm_1.0-11 DBI_1.0.0 Rcpp_1.0.2 plotrix_3.7-6

# [55] cmprsk_2.2-8 htmlTable_1.13.2 foreign_0.8-71 stats4_3.6.1 heplots_1.3-5 survey_3.36 glmnet_2.0-18 httr_1.4.1 htmlwidgets_1.3

# [64] RColorBrewer_1.1-2 lavaan_0.6-5 acepack_1.4.1 IsingFit_0.3.1 mice_3.6.0 pkgconfig_2.0.3 R.methodsS3_1.7.1 nnet_7.3-12 tidyselect_0.2.5

# [73] rlang_0.4.0 reshape2_1.4.3 polynom_1.4-0 munsell_0.5.0 cellranger_1.1.0 tools_3.6.1 cli_1.1.0 generics_0.0.2 IsingSampler_0.2

# [82] broom_0.5.2 fdrtool_1.2.15 knitr_1.25 zip_2.0.4 mitml_0.3-7 GeneNet_1.2.13 glasso_1.11 pbapply_1.4-2 nlme_3.1-140

# [91] quantreg_5.51 whisker_0.4 R.oo_1.22.0 smacof_2.0-0 xml2_1.2.2 compiler_3.6.1 rstudioapi_0.10 curl_4.2 png_0.1-7

# [100] huge_1.3.3 pbivnorm_0.6.0 stringi_1.4.3 Epi_2.38 eigenmodel_1.11 Matrix_1.2-17 nloptr_1.2.1 vctrs_0.2.0 parcor_0.2-6

# [109] pillar_1.4.2 lifecycle_0.1.0 data.table_1.12.2 corpcor_1.6.9 R6_2.4.0 latticeExtra_0.6-28 gridExtra_2.3 rio_0.5.16 codetools_0.2-16

# [118] polspline_1.1.16 boot_1.3-22 MASS_7.3-51.4 gtools_3.8.1 assertthat_0.2.1 rjson_0.2.20 withr_2.1.2 mnormt_1.5-5 multcomp_1.4-10

# [127] ppls_1.6-1.1 mgcv_1.8-28 parallel_3.6.1 hms_0.5.1 grid_3.6.1 rpart_4.1-15 minqa_1.2.4 carData_3.0-2 d3Network_0.5.2.1

# [136] lubridate_1.7.4 numDeriv_2016.8-1.1 base64enc_0.1-3 ellipse_0.4.1 #
